# The Massachusetts Veterinary Association*Read at a meeting of the above Association at Boston, Mass., May 7, 1884.

**Published:** 1884-07

**Authors:** F. S. Billings


### Art. XVIII.—THE MASSACHUSETTS VETERINARY ASSOCIATION.*

BY F. S. BILLINGS, V.S.

Mr. President and Gentlemen :

In asking me to initiate the work for which we have come together, you have thrown a degree of responsibility upon my shoulders that might well have been shared by others. The selection of a subject conformable to the purpose of an introductory essay before the members of this Association has not been by any means an easy task.

I have decided to ask you to give your attention to some of the work of this Association as suggested by the "Constitution" we are going to present to your consideration this evening.

What are we here for?

For what purposes are we forming this Association?

The "Constitution" we have to offer you opens something as follows :

"One of the purposes of this Association shall be to encourage collegiate feelings, not only among the *graduated* veterinarians of this State, but with the *graduated* men in our profession in other States."

You will observe that we start out with a new restriction, which has not been made either by the so-called "United States Veterinary Association" or many other such bodies.

This Association at once sets the future standard as to what shall constitute a veterinarian in Massachusetts.

It mastheads the flag of right, of honor, and of independence.

It says to the citizens of the "Bay State" that the days of the "cow-leech," "horse-doctor," empiric and quack must soon be among the "has beens," and that no man is entitled to be called "a veterinarian" unless he has "*graduated*" at some recognized veterinary school.

Clause 2d says : "That we are to do our utmost for the advancement of our profession, both as an association and indi-

---

* Read at a meeting of the above Association at Boston, Mass., May 7, 1884.

viduals." When we adopt this clause we have each solemnly undertaken many obligations which should have always been ours, but have not, I fear, with many of us.

The time seems to have come, or is fast approaching, when we shall be justified in speaking of a veterinary profession in the United States, of some kind or other. What it will be depends upon its members individually and collectively. Unless every individual veterinarian in the land feels that, aside from the imperative obligations due to himself and those he has dependent upon him, he has no less important ones toward the profession he has adopted, we shall make no advancement. The sporadic work of a few individual members of our profession will avail us nothing. It makes certain men altogether more prominent than the work thus far performed would justify, but does nothing for the elevation of the profession. Individual endeavors, when limited to sporadic or even continued exertions, will avail us but little. We must learn that *good work* can only result from the united endeavors of every one of us. "Pull, Pat; I pulled last," will not do for our motto. It must rather be "a long pull, a strong pull, and a pull altogether." We must learn that "a house divided against itself cannot stand." By taking the position that none but "*graduated*" men have the right to the title of "veterinarian," we have taken the fundamental and, in reality, most necessary step toward putting a solid foundation under our structure. Our house will not be built upon the shifting sands of ignorance and superstition, but rather upon the rock of right; upon which we shall plant the "Tree of Knowledge," and against which neither the waves of bigotry nor the tempests of quackery and fraud shall prevail.

We must stick to this principle, in order that our efforts may be rewarded by success.

We have not yet been able to make ourselves felt as a power in the land. Why?

Because we have never yet been represented by men of sufficient calibre to do any more than to endeavor most faithfully to represent their insignificant selves.

"*Self*" has been their only *devotion.*

This brings us to the consideration of another very important clause, viz.:

"Recognizing the intimate relations existing between various animal diseases and the public health, and the gradual extension which contagious and infectious diseases are acquiring among our domestic animals, this Association earnestly advocates the perfection of an organized veterinary police system in Massachusetts."

Gentlemen, here is our work! Never before in the history of Massachusetts have the times and circumstances been so propitious as now.

The same is true of the majority of the States in the Union.

The careless and unpardonable neglect of the representative of the United States Government at Portland, by which the foot and mouth disease gained a temporary footing on our shores, only once more endorses a fact which history has so often proved true; and that is, that empiricism is never to be depended upon. The countries of Europe discovered this fact over one hundred years ago. It cost them more millions to find this out than our national debt ever amounted to. It has probably cost this country an almost incomprehensible sum, could we know the value of the animals that have perished from preventible causes, even during this century. Empiricism struck the rock of adversity that time, gentlemen. Whatever other governments may think, whatever obstinacy and ignorance may still continue to do at Washington, members of the Massachusetts Veterinary Association, let us not be slow in proclaiming that this act is in no way connected with the veterinary profession of the United States.

It justifies us in our determination that none but "*graduated*" men should be considered veterinarians.

The time has come to begin organizing a veterinary police and hygienic system in Massachusetts. The days of "cattle commissions" should be numbered in the land. The "Bay State" should, to be true to her record, be the first to recognize the necessity of, and to organize such a system.

What does it mean, gentlemen? It means work for us, on the one hand, and the protection of human life, on the other.

We want our best man at the helm.

We want no politician, no wire-puller, no fellow with more suavity of manner than either brains or devotion to our profession. Mere education won't do, either. The only man that can fill such a place is that one among us who unites the requisite educational and practical abilities with the best executive ability.

But that is not all. The one great "gift" has not been mentioned. We want a man as State veterinarian in Massachusetts that can forget that he lives, save only as our profession lives in him. No man who desires the position for himself is worthy our notice. We want work. Aye, gentlemen, we are bloodthirsty! We want the devotion of a life, if necessary, to bring our profession to the place it *must*, not *should*, occupy in public estimation.

Massachusetts, the State so proud of its support of education, must no longer insult us by flaunting empiricism in our faces. We must not take our hands from the plow until we have a State veterinarian at our capital, a county veterinarian in every county, district veterinarians wherever necessary, veterinarians as market inspectors, and veterinarians at all our slaughter-houses.

It would seem as if we wanted to "scoop the treasury" when we say that every one of these men must receive honorable recompense from the public funds.

The laborer is worthy of his hire. Let us see what we have to offer for the remuneration we have a right to demand. The public may also demand something of us. What?

1. That every hog offered for sale in Massachusetts is free from trichinosis, and every animal slaughtered fit for human food.

2. That every milch-cow in the State is under our supervision, and registered. That every one of these cows is hygienically housed and fed, and absolutely free from disease.

This means, to lessen the chances of tuberculosis and "summer complaint" among the babies and children fed on the bottle.

3. The most perfect control possible of our animals, with reference to contagio-infectious diseases.

This means protection of human life from hydrophobia, anthrax, glanders and the like, on the one side, and the protection of every man's animals from the attack of such diseases, on the other.

Such an institution means organized work, organized observations, the accumulation of valuable statistics. Certain results can only follow comprehensive, systematic, and well organized work.

It is the sum of all men's work that makes up our knowledge, not that of any one man, however brilliant he may have been.

We must attain this end, or our work will have been in vain. It is for this purpose that we have, in all truth, formed this Association.

Collegiate intercourse, the reading of papers and discussions, even the endeavor to ethically regulate the practice of our profession, *among graduates*, are all but means towards this one great end.

'Tis a noble aim, gentlemen!

Nobler than the world knows of at present.

It is the saving and promulgation of human and animal life which we are aiming at.

Our medical colleagues have not a grander or nobler field of devotion.

We cannot obtain this end except by unity of purpose. We must work as one man! The petty jealousies, encouraged by the schools, must be smothered. Our Alma Maters are behind us! Things of the past! The present only is with us! To-day we must prepare for the morrow! It matters not from what school we may have come, we must forget that it asserted it "had more graduates in successful practice than any other;" or that "it was the only school legally authorized to give the diploma of Veterinary Medicine in the United States." We must not make such *lies* our own, but rather feel such humbuggery is behind us, and that being free, having our passport to the profession, we have entered upon the roadway of active life, and become honorable members of an honorable profession.

Neither the Alma Mater nor the diploma, nor even the education makes the man.

He makes himself! They are his tools! "By your good works shall ye be known!"

We have other work before us and I must draw my paper to a close, but I cannot do so without asking your attention for a few moments, to an equally important subject to any of those so cursorily touched upon in the preceding remarks.

To this constitution we have supplemented a

CODE OF ETHICS.

By that we mean *Medical Morals.* While we would not countenance rascality in any member of this Association, yet the code of ethics of a medical society has nothing to do with many individual acts of its members that are not in accord with the most stringent rules of social or religious morality; it has to do most emphatically with what is known as professional morality.

Gentlemen, many of you may have heard of the Irish tailor who, dragging his coat behind him, went out on the street crying, "Who'll thrid on the tayles o' mi coat. I'm mouldhering fur a batin'."

Well, I am somewhat in that condition to-night. During our several meetings the fires have been slumbering, but it is time the throttles were opened and steam let on. Who among us did not at first feel glad to hear that the honored and oldest educational institution in New England was going to inaugurate a veterinary school or department? Is there a gentleman here to-night, save those connected with this school, *that dare affirm he rejoices in its existence?*

We have a clause in our "Code of Ethics" which reads as follows:

"The business relations of the veterinarian to his profession and the public:

"The professional success of a practitioner depends upon qualities connected with his moral character, his scientific attainments, and also his industry and business talent. The kind of competition which might be considered honorable in business cannot exist between veterinarians without diminishing their usefulness and lowering the standard of the veterinary profession."

If these are to be the sentiments that are to be the rule among us, by which we are to be judged and are to judge one another, then how much more are they applicable to the veterinary institutions of the land, especially that of our own State, and the veterinarians attached to them, than to the ordinary members of the profession.

The school is the nursery of the profession. Its professors its moral and scientific lights. Students should be able to look back upon their Alma Mater with pride, and to their teachers with respect.

How is it with Harvard? Does her school conform to the spirit of the above rule of ethics? On the contrary, is it not conducted in a manner directly opposed to it, when they agree to treat, for ten dollars per year, an unlimited number of horses, at the hospital, for the small sum of one dollar extra per day; when in addition to that, they agree to examine ten horses per year, for soundness, and to give any amount of advice, to this same subscriber, at the hospital, free of any extra charge?

When, in addition to this, they offer to treat the animals of non-subscribers as follows:

Board, treatment, and medicine for sick horses, per day, $2.00; board, care, and medicine for surgical cases (horses), per day, $1.00; board, treatment, and medicine for dogs, per day, .50; board, treatment, and medicine for cattle, per day, $1.00; examination and advice at hospital, $1.00.

Now, gentlemen, what have you to say to such ethics as that? What have you to say of the ethical deportment of colleagues that are willing to accept an appointment as teacher at such an institution? At an institution, gentlemen, that, like its London prototype, first seeks students, then charges them heavy fees for tuition, and finally turns them loose to starve on a practice ruined by this profession-destroying subscription plan?

You will be told, gentlemen, that "they must have a clinic," that "they did not know how to get one otherwise."

All bosh, all bosh!

The great schools at Paris and Berlin (and every other Continental school) can treat their eleven or twelve thousand patients a year without any such nonsense. Philadelphia is going to do it, and Harvard could do it, but she wont.

What cares Harvard about our profession, gentlemen?

She has given her answer in her actions.

Not one iota!

What care her veterinary professors about a profession they are doing their utmost, heedlessly—and headlessly, perhaps—to demoralize and degrade? How can we have fraternal feelings towards these gentlemen when they persist in such unprofessional practices? Is it honorable for them to use the advantages which their position has given them to gain private practice?

Can they justify their advertising that "calls for outside visits will be attended to at any time, day or night," by any Code of Medical Ethics?

We want them to succeed. Every veterinarian, every citizen of Massachusetts, that takes an honest pride in our public institutions—and which of them is greater than Harvard?—wishes to see this venture successful.

Gentlemen, if you do not know it, it is time that you all learned that merely practical reputations are as ephemeral as man's life. It is only what we do, that has in it lasting benefit to our race that lives after us. We should be judged by our knowledge rather than by our success in practice, and these gentlemen will find that the verdict of history, which is always just, will be the same as has been given of the London school: "A dog in the manger, that neither did anything itself, but opposed progress when proposed by others."

We can only speak for ourselves. For myself I can honestly say to you, that neither offers of money nor position will ever lead me to knowingly demean myself by the support of any principle or act liable to have an injurious influence upon our profession. It may be my misfortune, but I have not that respect and reverence for my Alma Mater, or my country, that most people seem to think a virtue. I can see errors in my own mother, and am not afraid to point them out. I can see my own and am not afraid to have them pointed out, and have nothing but respect for my critic. Nothing is sacred but the truth.

Let me assure you all, let me assure the gentlemen from Harvard Veterinary School, that personality, ill-feeling, or jealousy, does not enter my mind in this discussion.

When we have acquired our diplomas, we have entered upon new duties, new responsibilities. We have assumed obligations to one another, to our profession. We must each so deport ourselves as not to injure the standing of the latter or retard its progress.

I am trying to represent the profession. I live only that the profession may live in me. My life work is the advancement of our profession. My methods may be wrong. Time will render a just verdict on them. They are the outcome of years of study of this one question: "the foundation of the world's veterinary institution, and the lessons they teach." Perhaps I have read them erroneously, but I think not.

To me the "subscription plan" is the greatest curse that can come upon us. It is unprofessional in the highest sense. I cannot comprehend how any man that means to be true to his profession, how an American veterinarian that thinks himself able to teach—and no man is fitted for that high office that has not critically studied the history of our profession—could be found willing to connect himself with any school conducted under this plan.

We want this school to be successful. We must make it so. We can only do it by publicly and privately opposing this plan. We shall not be true to ourselves, or to our country and profession, if we do not. How are we to do it?

By earnestly endeavoring to keep students away from it while the plan is its foundation stone.

To the students we would also say, that if they have any idea of the professional duties which they must assume on their graduation, that they cannot and should not help support such a school as this. One that, the moment they graduate, becomes their opponent in practice, not on the honorable terms of competition based on ability, but on the most dishonorable principles that the human mind can conceive of.

We certainly cannot look upon such an institution as an honorable school, nor can one of us recommend it to a brother or a friend while so conducted.

The responsibility is upon our shoulders. Our colleagues all over the land will be found in earnest sympathy with us.

Stop! Think!

There is but one golden rule of life: "Do as you would be done by."

We vigorously condemn any cutting of prices in each other. Shall we be any less lenient to the schools from whence our future colleagues are to come?

If Harvard has an ethical right to thus cut prices, have not her graduates the same?

What principles to bring up youths under!

The medical profession would not put up with such action on the part of the medical school for an instant.

Shall we be less honorable?

As we act in this matter so will our colleagues in other States judge us.

We have then to decide to-night one of the great questions of importance.

Will we give our professional and moral support to a public institution, for the education of veterinarians, founded upon the subscription plan?

Can we do this and be honest to the responsibilities we assumed when we entered the profession?

Gentlemen, I am done! The verdict is in your hands. You are judge and jury in this case. Reflect calmly, but judge ye righteously! The veterinary profession of America awaits your answer.

